# ﻿A new *Pseudophoxinus* species (Teleostei, Cypriniformes, Leuciscidae) from the upper Jordan River basin (Israel) with comments on the status of a few other congeneric species

**DOI:** 10.3897/zookeys.1249.154110

**Published:** 2025-08-25

**Authors:** Menachem Goren, Tamar Feldstein-Farkash

**Affiliations:** 1 The Steinhardt Museum of Natural History, Tel Aviv University, Tel Aviv, Israel Tel Aviv University Tel Aviv Israel; 2 School of Zoology, Faculty of Life Sciences, Tel Aviv University, Tel Aviv, Israel Tel Aviv University Tel Aviv Israel

**Keywords:** COI, DNA barcoding, freshwater fish, integrated taxonomy, minnows, new species, phylogenetic analysis

## Abstract

A taxonomic reassessment of *Pseudophoxinus* populations in the upper Jordan River basin has revealed that specimens previously identified as *Pseudophoxinus kervillei* actually represent an undescribed species. In addition, earlier taxonomic revisions have shown that *P. kervillei* is a junior synonym of *P. libani* and should no longer be regarded as a valid species. In this study, we formally describe the newly recognized species as *Pseudophoxinus galilaeus***sp. nov.***Pseudophoxinus galilaeus***sp. nov.** is characterized by 39–44 scales along the mid-lateral row, 13–17 pored lateral line scales, and 20–23 predorsal scales. It has 4–5 gill rakers on the lower arch of the first gill, with two being notably short. The species possesses 33–34 vertebrae; its dorsal fin originates at vertebrae 12 or 13, and its anal fin commonly originates at vertebra 19, occasionally extending to vertebra 20 or 21. *Pseudophoxinus galilaeus***sp. nov.** inhabits ponds, lakes, and rivers with slow to moderate currents. A unique DNA barcoding signature (mtDNA COI) revealed that it differs from any other previously bar-coded *Pseudophoxinus* species. In phylogenetic analyses, it clustered with the *Pseudophoxinus* species from neighboring countries in the Levant region, suggesting a common ancestor for these species. This analysis shows that sequences of *P. kervillei* from Turkey differ from *P. libani* from Lebanon and Syria. Further morphological examination is needed to determine the status of the species.

## ﻿Introduction

The genus *Pseudophoxinus* Bleeker, 1860 (Cypriniformes, Leuciscidae) comprises 27 valid species. However, Froese and Pauly (2025) listed 29 valid species, while Fricke et al. (2025) reassigned two of these species to other genera. The following nominal species have been reported from the Levant (Israel, Syria, Lebanon, Jordan): *P. kervillei* (Pellegrin, 1911), *P. libani* (Lortet, 1883), *P. syriacus* (Lortet, 1883), *P. zeregi* (Heckel, 1843), *P. drusensis* (Pellegrin, 1933) (*Pseudophoxinus hasani* Krupp, 1992, known only from Nahr Marqiya (northwestern Syria), and *Pseudophoxinus turani* Küçük & Güçlü, 2014, reported from İncesu Spring (Orontes drainage, Turkey) (Bariche and Freyhof 2016; Çiçek et al. 2023, 2024). Two of the species have been reported from Israel: *Pseudophoxinus drusensis* from the Golan Heights (Goren 1972, as *Pseudophoxinus zeregi drusensis*) and *Pseudophoxinus kervillei* from Lake Hula, Upper Galilee (Tortonese 1938). The species *P. kervillei* was described by Pellegrin (1911, 1923) from the Orontes River, near Homs (Syria) and based on 13 small specimens, with a total length ranging from 14 to 44 mm.

Tortonese (1938) was the first to report the presence of this species in the Jordan River basin. He examined nine specimens from Lake Hula (upper Jordan River basin) with standard lengths of 24–50 mm. He identified the fish as *P. kervillei* but noted that some meristic counts and body proportions differed from Pellegrin’s specimens, particularly in eye diameter, the number of pored lateral scales, the length of the pectoral fin, and the presence of a black blotch at the base of the caudal fin. Norman and Trewavas (1938: 556) also reported the presence of this fish in Lake Hula, though they were uncertain of its identity and listed it as “*Phoxinellys* (? kervillei Pellegrin)”. Steinitz (1951), who compared a few specimens from Lake Hula in Israel with the species’ original description by Pellegrin (1911), noted differences in body proportions. He also reported slight differences from the specimens reported by Tortonese (1938) from Lake Hula. In 1953, Steinitz (p. 213) wrote: “Recently we have given a short account of the species with emphasis on deviations of our material from that reported by previous authors.” Goren (1969, 1975), who compared populations from the Hula Valley with eight specimens from Charka Spring, a tributary of the Orontes River, also reported differences between the two populations, including skeletal variations. Krupp and Schneider (1989: 378), who studied the fishes of the Jordan River basin, stated: “However, we collected new specimens at the type locality, and there is no doubt as to the identity of this species.” Bariche and Freyhof (2016), based on morphological and molecular characters (COI) analysis, concluded that *Pseudophoxinus kervillei* is a junior synonym of *P. libani* (Lortet, 1883).

The present study was initiated following a survey of freshwater fish in Israel to define sites important for conservation (Tadmor-Levi et al. 2023). The survey included a comprehensive phylogenetic analysis of all the freshwater fish species in Israel. The study revealed that *P. kervillei* in Israel belongs to a distinct, yet undescribed species of *Pseudophoxinus* spp. Using an integrated approach that combines morphological and molecular methods, we describe the population of the upper Jordan River basin as a new species.

## ﻿Methods

Fish specimens were obtained from the Steinhardt Museum of Natural History at Tel Aviv University.

Counts and measurements followed the methods of Kottelat and Freyhof (2007).

### ﻿Abbreviations

**SMNHTAU-P** – Fish collection of the Steinhardt Museum of Natural History; **FL** – Fork length; **SL** Standard length; **TL** – Total length.

### ﻿Sequence analyses

Phylogenetic analyses were performed for *Pseudophoxinus* species from the Levant inhabiting water systems in Israel, Lebanon and western Syria. The barcoding region of the COI mitochondrial gene was used for reconstructing the phylogenetic tree. COI sequences were downloaded from GenBank and aligned with MAFFT v. 7.304 (Katoh and Standley 2013) using E- INS-i parameters. The maximum likelihood tree was reconstructed using IQ-TREE 2 (Minh et al. 2020).

Branch support was evaluated with 1000 repeats of ultrafast bootstrap (UFBoot; Minh et al. 2013). The evolutionary divergence between sequence pairs of species was estimated in MEGA 11 (Tamura et al. 2021). The rate variation among sites was modeled with a gamma distribution (shape parameter = 1). Sequences used for the molecular analyses are listed in Suppl. material 2. In addition, a comprehensive phylogenetic tree of COI sequences from all the species of the genus *Pseudophoxinus* is provided in the Suppl. material 1.

## ﻿Results

### ﻿ Pseudophoxinus galilaeus sp. nov.

Taxon classificationAnimaliaCypriniformesLeuciscidae

E5587D73-11AD-5D9F-87A3-65758904F94A

https://zoobank.org/7ABB4D05-D9DC-4E1D-866B-32377F624D3E

Figs 1 , 2 , 3 , 4

Phoxinellus kervillei (non Pellegrin, 1911) Tortonese 1938; Steinitz 1951, 1953, 1954.
Pseudophoxinus [sic] kervillei (non Pellegrin, 1911) Goren 1975, 1983; Ben Tuvia 1978; Krupp and Schneider 1989; Goren and Ortal 1999; Gasith et al. 2000; Ostrovsky et al. 2014; Tadmor-Levi et al. 2022; Tadmor-Levi et al. 2023; Çiçek et al. 2023.
Phoxinellus zeregi (non Heckel, 1843) Lortet 1883.

#### Material examined.

***Holotype*.** • SMNHTAU-P.17856, TL: 82.0 mm, SL69.6 mm, collected in Dan River, 15 September 1988 by M. Goren (Fig. 2). ***Paratypes***: • SMNHTAU-P.10667, 5 specimens, TL: 38.8–82.3 mm, SL: 31.3–69.5 mm, same data as for holotype. • SMNHTAU-P.242, 3 specimens, TL: 78.8–94 mm, SL: 63.2–75.6 mm. Collected at Hula Lake, 7 August 1954, (probably by H. Mendelssohn). The Lake drained during 1954–1958 (southwestern part kept as a nature reserve).

**Figure 1.**
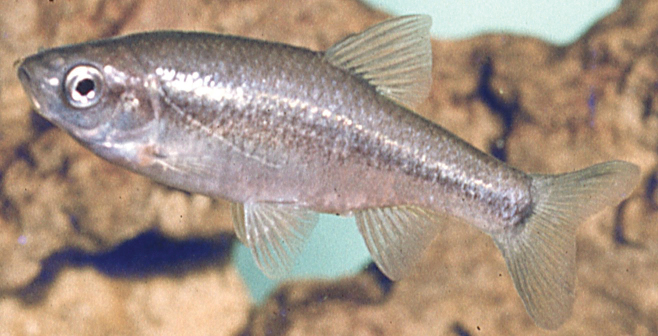
*Pseudophoxinus galilaeus* sp. nov. An aquarium picture (1968, photo A. Subb).

**Figure 2.**
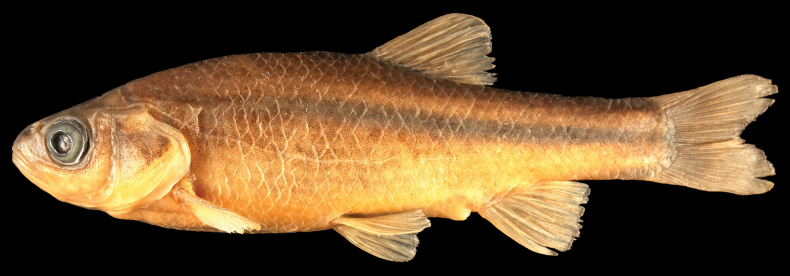
Holotype of *Pseudophoxinus galilaeus* sp. nov. SMNHTAU-P.17856 (Photo: O. Rittner.)

#### Additional material.

• SMNHTAU- P.3021, 108 specimens, TL: 25.6 mm – 43.2 mm, SL: 21.0–75.6 mm, collected by M. Goren, Ein Einan (tributary to Hula Nature Reserve), 12 June 1968; • SMNHTAU-P.7540 13 specimens, TL: 25.6–43.2 mm, SL: 21.0–75.6 mm, collected by M. Goren, Dan River 6 September 1979; • SMNHTAU-P.11568 33 specimens, TL: 40.5–51.0 mm, SL: 32.7–41.5 mm, collected by D. Hisdai Meshushim River (tributary of Lake Kinneret) March 1988; • SMNHTAU-P.3027, 4 specimens, TL: 43.5–47.7 mm, SL: 34.9–38.6 mm, collected by M. Goren, Hula Nature Reserve, 20 October 1968; • SMNHTAU-P.13300, 3 specimens, TL: 48–54 mm, SL: 40–46 mm Hula Nature Reserve, 28 November, 2007; BMNH 1968.12.13.343–350, 8 specimens TL: 36–44 mm, SL: 29–35 mm, Charka spring (West side of Ghab, connects to Orontes River, Syria), 27 November 1957.

#### Samples used for phylogenetic analysis.

• SMNHTAU-P.15771, TL 60 mm, SL 50.1 mm, collected by E. Elron, Einot Gonen, 16 May, 2015. • SMNHTAU-P.15826, TL 70 mm, SL 59 mm, collected by E. Elaron, Agmon Ha’Hula, 14 July, 2015. • SMNHTAU-P.15800, TL 47 mm, SL 38 mm, collected by E. Elron, Dan River, 21 July 2015. • SMNHTAU-P.14406, TL 41 mm, SL 33 mm, collected by M/ Goren at the mouth of Daliyot River, 9 December 2011. • SMNHTAU-P.14428, TL 79 mm, SL 67 mm, collected by M. Goren, Meshushim River, 9 December 2011. • SMNHTAU-P.14421 TL 54 mm, SL 46 mm, collected by M. Goren, Meshushim River, 9 December 2011. The samples used for phylogenetic analysis were the same as those analyzed in the study by Tadmor-Levi et al. (2023).

#### Diagnosis.

A species of *Pseudophoxinus*, characterized by 39–44 longitudinal scale series along the body, 13–17 pored lateral line scales, 20–23 predorsal scales, 4–5 gill rakers on the lower limb of the first gill arch (with 2–3 rudimentary rakers at the rear) and 30–32 vertebrae.

#### Description.

A small fish, rarely exceeding 9.0 cm in length (TL). The lateral line is incomplete and never extends to a vertical line from the anus.

Fins: The front of the dorsal fin is behind a vertical line from the base of the pelvic fin; The front of the anal fin is behind the rear end of the dorsal-fin base. Dorsal fin with 7–9 segmented rays. Anal fin with 7–8 segmented rays. Pectoral fin with 13–14 segmented rays. Pelvic fins with 7 segmented rays (6 in a single examined specimen); Caudal fin Forked, with 20–24 segmented rays. The shortest caudal ray (mid-ray) is approximately half the length of the longest ray.

Scales: The body is covered with cycloid scales, while the head is naked. Longitudinal scale series: 39–44; Transversal scale series: 13–15 (counted from the insertion of the dorsal fin backward); Predorsal scales: 20–23; Pored lateral line scales: 13–17; In some specimens, 2–3 unpored scales interrupt the curve of the pored lateral line scales.

Vertebrae: The total number of vertebrae is 30–32 (with the first vertebra bearing a neural spine counted as the third vertebra). Dorsal fin originates above vertebrae 10 or 11, while the anal fin originates below vertebrae 19 to 21 (vertebral count data from Goren 1969).

Pharyngeal teeth: 4–5.

Gill rakers on lower limb: 4–5, the 2–3 rear are rudimentary.

**Figure 3.**
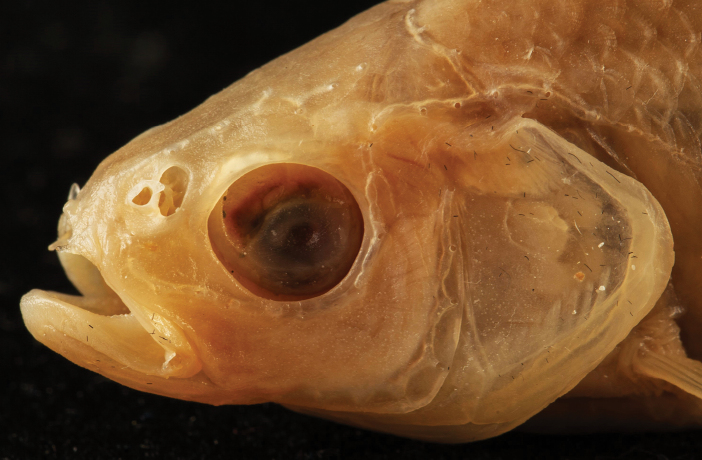
Profile aspect of the head of *P. galilaeus*. (Photo: O. Rittner).

Nostrils: The posterior nostril is an oval pore located above the front of the eye. The anterior nostril has a short tubular pore (Fig. 3).

**Figure 4.**
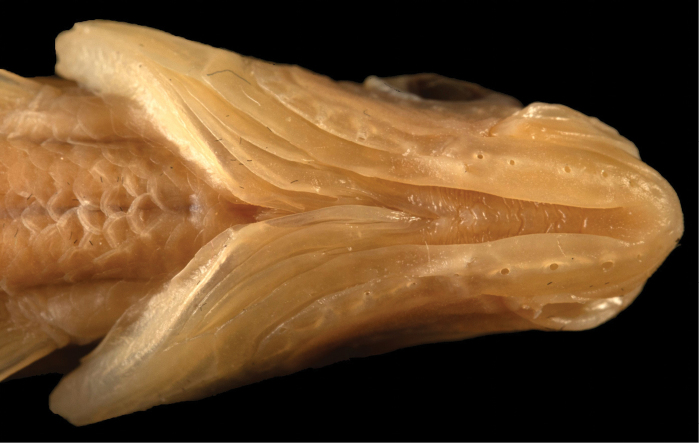
Ventral aspect of the head of *P. galilaeus*. (Photo: O. Rittner).

Cephalic sensory system: The lateral line canal extends from the pored scales to the upper margin of the operculum, then curves around the lower margin of the eye to the snout. A branch of this canal is located on the back of the head, just in front of the predorsal scales. A second canal runs from the upper margin of the preoperculum to the lower jaw, extending slightly before the tip of the jaw (Figs 3, 4).

Additionally, two pored canals run across the top of the head, extending from above the first nasal pore through the interorbital space to the back of the head.

***Meristic counts and body proportions*** of the holotype and paratypes are presented in Table 1. Additional data from non-type examined specimens are provided in the Suppl. material 3.

**Table 1.** Meristic counts and body proportions of the holotype and eight paratypes of *Pseudophoxinus galilaeus* sp. nov. SL = standard length; TL = total length.

Counts	Holotype	Paratypes		
		Range	Average	Standard deviation
No. of pored lateral line	14+3*	13–17	14.3	1.2
No. of scale series along the body	44	39–44	42.3	1.9
No. of scale in transversal series	15	13–15	13.7	0.8
Predorsal scales	20	20–23	20.9	1.5
Segmented dorsal fin	8	7–8	7.5	0.5
Segmented anal fin	7	7–8	7.8	0.5
Pectoral fin	14	13–14	13.5	0.5
Pelvic fin	7	6–7	6.9	0.4
Caudal fin rays	20	20–22	20.3	0.7
Gill rakers	4	4–5	4.6	0.5
Proportions (%)				
SL/TL	84.9	80.2–86.7	82.9	2.6
Fork length/TL	94.4	92.9–96	94.3	1.0
Shorter caudal ray/longest caudal ray	62.9	62.9–68.4	66.5	3.1
Head length of SL	27.0	27.1–33.7	28.6	1.9
Body depth of SL	27.3	25.4–31.3	27.7	3.0
Eye diameter of head length	27.7	26.5–32.7	26.9	2.1
Depth of caudal peduncle of SL	12.1	9.5–13.3	11.8	1.2
Length of caudal peduncle of SL	19.0	18.2–2104	19.5	1.0
Interorbital space of head length of SL	41.0	33.7–42.5	39.1	2.4
Longest dorsal ray of SL	19.0	11.6–24.8	18.9	4.9
Longest anal ray of SL	14.5	12.2–16.8	14.5	1.6
Pectoral-fin length of SL	12.5	13.3–19.3	14.9	2.1
Pelvic-fin length of SL	12.5	47.3–54.1	13.9	2.2
Distance snout – pelvic fin of SL	49.4	46.3–53.7	49.2	2.4
Distance snout – pectoral fin of SL	27.2	26–31.9	28.7	2.0
Distance snout – dorsal fin of SL	55.9	12.3–14.2	55.3	2.1
Distance snout – anal fin of SL	71.7	68.5–70.4	65.8	9.4
Length of dorsal-fin base of SL	12.4	11.6–13.4	12.3	0.6
Length of anal-fin base of SL	10.2	10.7–12.8	11.2	0.8

* Interrupted by 2 non-porous scales.

***Coloration*** (live specimen) . The body is silvery-matte with a distinct dark band running along the mid- body, from the upper margin of the operculum to the middle of the caudal base, where it ends in a dark blotch. The fins are transparent with fine dark pigmentation.

#### Habitat.

*Pseudophoxinus galilaeus* sp. nov. inhabits slow-moving streams or still waters among stones and aquatic plants. In fast-flowing streams, it seeks shelter near small natural dams, among stones, or within tree roots. Its distribution includes the upper Jordan Basin, ranging from the Dan and Snir Rivers to Lake Kinneret (Goren 1975; Krotman 2004).

##### ﻿Phylogenetic analysis of *Pseudophoxinus* species from the Levant

**Figure 5.**
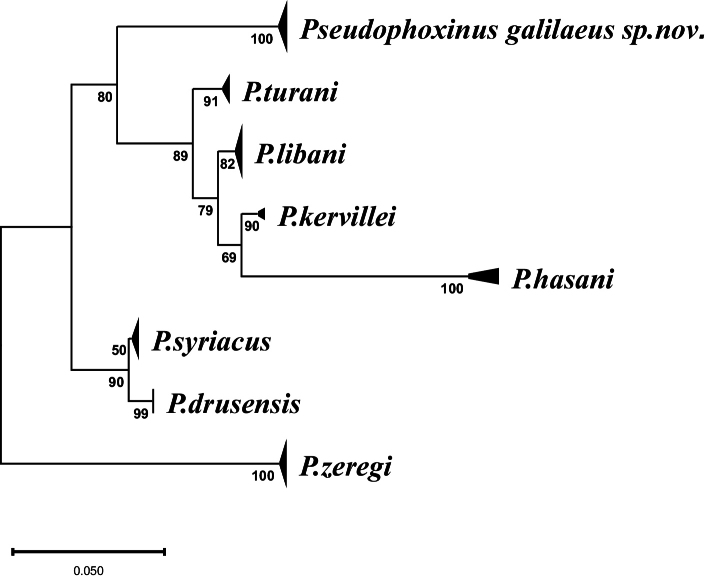
The phylogenetic relationships among Pseudophoxinus species from Israel and neighboring water systems. The maximum likelihood tree was reconstructed in IQtree2 using 48 sequences with 545 nucleotide sites (See Suppl. material 2 for sequence details).

In this study, we expanded the phylogenetic analysis to include additional *Pseudophoxinus* species from the Levant that were not included in Tadmor-Levi et al. (2023). The phylogenetic relationships among *Pseudophoxinus* species are presented in Fig. 5. The genetic distances between the newly identified species and other examined species are shown in Table 2. In this analysis, the lowest genetic divergence calculated was 7% between *P. galilaeus* and the *P. syriacus*/*P. drusensis* complex.

**Table 2.** Mean genetic distances between the species clusters as defined by the maximum likelihood phylogenetic analysis. The number of base differences per site calculated by averaging over all sequence pairs between groups is shown. The rate variation among sites was modeled with a gamma distribution (shape parameter = 1). This analysis involved 48 nucleotide sequences and a total of 545 positions. All ambiguous positions were removed for each sequence pair (pairwise deletion option). The lower left values are the mean distances calculated using the p-distance model, and the upper right values were calculated using the K2P model. The bold values are within-group distances. Evolutionary analyses were conducted in MEGA11.

	*P. galilaeus* sp. nov.	*P. syriacus* /*drusensis*	*P. drusensis* (Israel)	*P. turani*	*P. libani*	*P. kervillei*	*P. hasani*	*P. zeregi*
*P. galilaeus* sp. nov.	0.0027	0.0781	0.0846	0.0803	0.0831	0.0840	0.1189	0.1278
*P. syriacus* /*drusensis*	0.0697	0.0034	0.0114	0.0684	0.0683	0.0626	0.1117	0.1083
*P. drusensis* (Israel)	0.0748	0.0112	0.0000	0.0705	0.0751	0.0705	0.1156	0.1153
*P. turani*	0.0708	0.0612	0.0629	0.0052	0.0261	0.0336	0.0880	0.1294
*P. libani*	0.0730	0.0614	0.0669	0.0250	0.0028	0.0211	0.0824	0.1194
*P. kervillei*	0.0739	0.0566	0.0632	0.0318	0.0203	0.0018	0.0750	0.1205
*P. hasani*	0.0993	0.0941	0.0972	0.0769	0.0724	0.0669	0.0061	0.1392
*P. zeregi*	0.1064	0.0924	0.0975	0.1069	0.1007	0.1015	0.1139	0.0024

## ﻿Discussion

In a checklist of freshwater fishes of the Middle East, Çiçek et al. (2024) listed 27 species assigned to the genus *Pseudophoxinus*, all of which are endemic to the region. Most of these species have a restricted geographic distribution, as also shown by Küçük et al. (2013, 2024) and Küçük and Güçlü (2014). In Israel and adjacent regions, four valid species have been identified (Bariche and Freyhof 2016). They considered *P. kervillei* (Pellegrin, 1911) as a junior synonym of *P. libani* (Lortet, 1883). They also report variation in meristic counts among fish populations of *P. libani* from different water systems. In the present study, we compared some meristic counts of the four populations with those of *P. galilaeus* (Table 3) and found that the new species has the lowest number of longitudinal and transverse scales. A statistical test on the number of scales in the mid-lateral row between *P. libani* fishes from the Yammouneh Basin (YB), the Litani River (LR), and the Orontes River (OR) (data sourced from Bariche and Freyhof 2016), with the type specimens of *P. galilaeus* (PG) revealed a high level of significance of the differences between *P. galilaeus* and the various populations of *P. libani* (YB vs. PG p-value: 3.96e^-06^; LR vs. PG – p-value: 1.63e^-09^; OR vs. PG - p-value: 7.72e^-07^).

**Table 3.** A comparison of meristic counts among species of *Pseudophoxinus* species from Israel and neighboring countries. LL- number of scales in mid-lateral row; PS - number of pored scales in lateral line; TR- transverse scale series; D - number of branched dorsal-fin rays; A - number of branched anal-fin rays; P - number of pectoral-fin rays; V - number of pelvic-fin rays; GR - number of gill rakers (lower limb). Sources of information: (1) Barich and Freyhof 2016; (2) Goren 1972; (3) Lortet 1883; (4) Heckel 1843.

	LL	PS	TR	D	A	P	V	GR
*P. libani* (1)	42–56	6–19	14–21	6.5–8.5	5.5–7.5	11–15	6–7	4–7
*P. syriacus* (3)	48–49			10	8	14	7	
*P. zeregi* (4)	57–66	11–12	20	7	6	12	6	
*P. drusensis* (2)	53–60		20–21	7	6	12	4	
*P. galilaeus* sp. nov.	39–44	13–17	13–15	7–8	7–8	13–14	6–7	4–5

A comparison of certain skeletal elements was conducted by Goren (1969, 1975). He examined eight specimens of *P. kervillei* from Charka Spring (a tributary of the Orontes River, Syria) with specimens from Upper Galilee (Ein Einan), and found that the Israeli specimens have fewer vertebrae, and their anal and dorsal fins originate closer to the head than those of the Charka Spring specimens (Table 4). Goren (1969) compared certain skeletal elements of eight *P. kervillei* specimens from Charka Spring (tributary of the Orontes River, Syria) with those from Upper Galilee (Ein Einan), and found that the Israeli specimens have fewer vertebrae and that their anal and dorsal fins originate closer to the head than in the Charka Spring specimens (Table 4). He also reported that the average proportion of the shortest caudal ray to the longest ray was 50.6% (±1.7) in the studied fish, while in fish from Charka Spring, it was 61.6% (±5.8). In both cases, these differences were significant (α < 0.05).

**Table 4.** A comparison of vertebrae number and origin of anal and dorsal fins between populations for Galilee and Orontes River. Charka spring specimens: British Museum of Natural History, Catalogue number: 1968.12.13.343-350); Galilee specimens: SMNHTAU-P.3021.

Locality	Number of fish	Number of vertebrae			Origin of dorsal fin above vertebra No:				Origin of anal fin under vertebra No:			
		32	33	34	10	11	12	13	14	19	20	21
Ein Einan	10	3	7		5	5				7	2	1
Charka spring	8		2	6			1	6	1	1	7	

In the molecular analysis of Levantine species, *P. drusensis* exhibits greater genetic variation between the populations in Israel and in Lebanon compared to the variation observed between *P. drusensis* from Lebanon and *P. syriacus* from Syria (Table 2). Based on this analysis alone, synonymization of the two species might be considered. These findings are also supported by the analysis of Tadmor-Levi et al (2023). However, morphological measurements indicate a significant distinction between the two species. The molecular analysis reveals a distinct cluster of *P. kervillei* sequences originating from the Orontes River in Turkey, which are clearly separated from the *P. libani–kervillei* cluster found in Lebanon and Syria (Fig. 5), with a genetic divergence greater than 2% (Table 2). Although morphological descriptions of the Turkish specimens are lacking (Perea et al. 2010), further investigations are necessary to assess whether *P. kervillei* represents a valid species.

In the case of the newly described species, molecular analysis provides strong support for the morphological analysis, revealing a significant genetic distance between *P. galilaeus* and its closely related species.

## Supplementary Material

XML Treatment for Pseudophoxinus galilaeus

## ﻿Additional information

### Use of AI

No use of AI was reported.

### Author ORCIDs

Menachem Goren https://orcid.org/0000-0003-1597-269X

Tamar Feldstein-Farkash https://orcid.org/0009-0007-9277-1417

### Data availability

All of the data that support the findings of this study are available in the main text or Supplementary Information.

## Supplementary materials

Supplementary material 1 Comprehensive phylogenetic analysis of Pseudophoxinus spp. Data type tiff https://binary.pensoft.net/file/1401459This dataset is made available under the Open Database License (http://opendatacommons.org/licenses/odbl/1.0/). The Open Database License (ODbL) is a license agreement intended to allow users to freely share, modify, and use this Dataset while maintaining this same freedom for others, provided that the original source and author(s) are credited.Menachem Goren, Tamar Feldstein-Farkash

Supplementary material 2 List of sequences of *Pseudophoxinus* sp. used for the reconstruction of a maximum likelihood phylogenetic tree based on the barcode sequences from the COI mitochondrial gene, and for the estimation of evolutionary divergence over sequence pairs between species Data type docx https://binary.pensoft.net/file/1401460This dataset is made available under the Open Database License (http://opendatacommons.org/licenses/odbl/1.0/). The Open Database License (ODbL) is a license agreement intended to allow users to freely share, modify, and use this Dataset while maintaining this same freedom for others, provided that the original source and author(s) are credited.Menachem Goren, Tamar Feldstein-Farkash

Supplementary material 3 Selected meristic counts and body proportions of non-type specimens of *P. galilaeus* Data type docx https://binary.pensoft.net/file/1401461This dataset is made available under the Open Database License (http://opendatacommons.org/licenses/odbl/1.0/). The Open Database License (ODbL) is a license agreement intended to allow users to freely share, modify, and use this Dataset while maintaining this same freedom for others, provided that the original source and author(s) are credited.Menachem Goren, Tamar Feldstein-Farkash
