# Supplementary figures and images for: ﻿A new Pseudophoxinus species (Teleostei, Cypriniformes, Leuciscidae) from the upper Jordan River basin (Israel) with comments on the status of a few other congeneric species
Source: Zookeys. 2025 Aug 25;1249:303–15. doi: 10.3897/zookeys.1249.154110 (PMC12402820; doi:10.3897/zookeys.1249.154110)

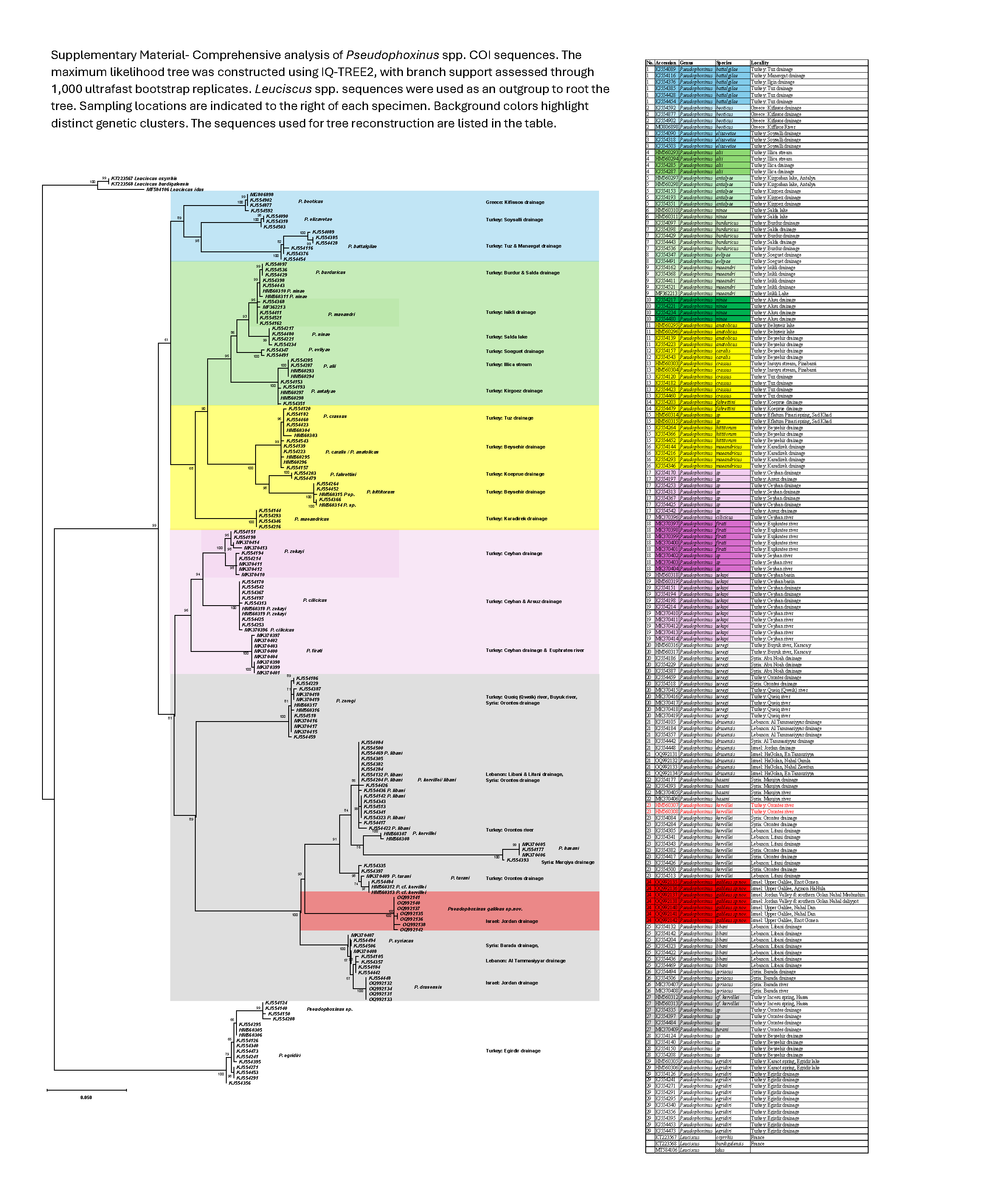

Supplement: Supplementary material 1 — Comprehensive phylogenetic analysis of Pseudophoxinus spp. [file zookeys-1249-303_article-154110__-s001.tiff]
