# Supplementary material for: ﻿A new Pseudophoxinus species (Teleostei, Cypriniformes, Leuciscidae) from the upper Jordan River basin (Israel) with comments on the status of a few other congeneric species
Source: Zookeys. 2025 Aug 25;1249:303–15. doi: 10.3897/zookeys.1249.154110 (PMC12402820; doi:10.3897/zookeys.1249.154110)
Supplement: Supplementary material 2 — List of sequences of Pseudophoxinus sp. used for the reconstruction of a maximum likelihood phylogenetic tree based on the barcode sequences from the COI mitochondrial gene, and for the estimation of evolutionary divergence over sequence pairs between species [file zookeys-1249-303_article-154110__-s002.docx]

**Table S1.** List of sequences of *Pseudophoxinus sp.* used for the reconstruction of a maximum likelihood phylogenetic tree based on the barcode sequence from the COI mitochondrial gene, and for the estimation of evolutionary divergence over sequence pairs between species.

| NCBI Accession | Species Cluster | GenBank annotation | Geo. Location /drainage | Reference | specimen voucher |
| --- | --- | --- | --- | --- | --- |
| OQ992143 | *Pseudophoxinus sp.nov.* | *Pseudophoxinus cf. kervillei* | Israel: HaHula | Tadmor-Levi et al. 2023 | Ps.ke0009 |
| OQ992135 | *Pseudophoxinus sp.nov.* | *Pseudophoxinus kervillei* | Israel: Jordan | Tadmor-Levi et al. 2023 | SMNHTAU:P.15771.1 |
| OQ992136 | *Pseudophoxinus sp.nov.* | *Pseudophoxinus kervillei* | Israel: Jordan | Tadmor-Levi et al. 2023 | SMNHTAU:P.15826.2 |
| OQ992140 | *Pseudophoxinus sp.nov.* | *Pseudophoxinus kervillei* | Israel: Jordan | Tadmor-Levi et al. 2023 | SMNHTAU:P.15800.2 |
| OQ992141 | *Pseudophoxinus sp.nov.* | *Pseudophoxinus kervillei* | Israel: Jordan | Tadmor-Levi et al. 2023 | SMNHTAU:P.15800.1 |
| OQ992142 | *Pseudophoxinus sp.nov.* | *Pseudophoxinus kervillei* | Israel: Jordan | Tadmor-Levi et al. 2023 | SMNHTAU:P.15771.2 |
| OQ992137 | *Pseudophoxinus sp.nov.* | *Pseudophoxinus kervillei* | Israel: Sea of Galilee | Tadmor-Levi et al. 2023 | SMNHTAU:P.14406 |
| OQ992138 | *Pseudophoxinus sp.nov.* | *Pseudophoxinus kervillei* | Israel: Sea of Galilee | Tadmor-Levi et al. 2023 | SMNHTAU:P.14428 |
| OQ992139 | *Pseudophoxinus sp.nov.* | *Pseudophoxinus kervillei* | Israel: Sea of Galilee | Tadmor-Levi et al. 2023 | SMNHTAU:P.14421 |
| KJ554448 | *P.drusensis* | *P.drusensis* | Israel: Jordan | Geiger et al. 2014 | ZFMK:FSJF:2181 |
| OQ992131 | *P.drusensis* | *P.drusensis* | Israel: Gamla | Tadmor-Levi et al. 2023 | SMNHTAU:P.14308 |
| OQ992132 | *P.drusensis* | *P.drusensis* | Israel: Gamla | Tadmor-Levi et al. 2023 | SMNHTAU:P.14311 |
| OQ992134 | *P.drusensis* | *P.drusensis* | Israel: Gamla | Tadmor-Levi et al. 2023 | SMNHTAU:P.14319 |
| OQ992133 | *P.drusensis* | *P.drusensis* | Israel: Zavitan | Tadmor-Levi et al. 2023 | SMNHTAU:P.14306.2 |
| KJ554105 | *P.syriacus* | *P.drusensis* | Lebanon: Al Tammasiyyar | Geiger et al. 2014 | ZFMK:FSJF:1221 |
| KJ554184 | *P.syriacus* | *P.drusensis* | Lebanon: Al Tammasiyyar | Geiger et al. 2014 | ZFMK:FSJF:1221 |
| KJ554357 | *P.syriacus* | *P.drusensis* | Lebanon: Al Tammasiyyar | Geiger et al. 2014 | ZFMK:FSJF:1221 |
| KJ554494 | *P.syriacus* | *P.syriacus* | Syria: Barada | Geiger et al. 2014 | ZFMK:FSJF:1319 |
| KJ554506 | *P.syriacus* | *P.syriacus* | Syria: Barada | Geiger et al. 2014 | ZFMK:FSJF:1319 |
| MK370407 | *P.syriacus* | *P.syriacus* | Syria: Barada | Geiger, Unpublished | FSJS-DNA-1319 |
| MK370408 | *P.syriacus* | *P.syriacus* | Syria: Barada | Geiger, Unpublished | FSJS-DNA-1319 |
| KJ554177 | *P.hasani* | *P.hasani* | Syria: Marqiya | Geiger et al. 2014 | ZFMK:FSJF:1330 |
| KJ554393 | *P.hasani* | *P.hasani* | Syria: Marqiya | Geiger et al. 2014 | ZFMK:FSJF:1330 |
| MK370405 | *P.hasani* | *P.hasani* | Syria: Marqiya | Geiger, Unpublished | FSJS-DNA-1330 |
| HM560307 | *P.kervillei* | *P.kervillei* | Turkey: Orontes | Perea et al. 2010 | MNCN AT5315 |
| HM560308 | *P.kervillei* | *P.kervillei* | Turkey: Orontes | Perea et al. 2010 | MNCN AT5316 |
| KJ554426 | *P.libani* | *P.kervillei* | Lebanon: Litani | Geiger et al. 2014 | ZFMK:FSJF:2151 |
| KJ554513 | *P.libani* | *P.kervillei* | Lebanon: Litani | Geiger et al. 2014 | ZFMK:FSJF:2151 |
| KJ554084 | *P.libani* | *P.kervillei* | Syria: Orontes | Geiger et al. 2014 | ZFMK:FSJF:1235 |
| KJ554417 | *P.libani* | *P.kervillei* | Syria: Orontes | Geiger et al. 2014 | ZFMK:FSJF:1243 |
| KJ554142 | *P.libani* | *P.libani* | Lebanon: Libani | Geiger et al. 2014 | ZFMK:FSJF:2154 |
| KJ554204 | *P.libani* | *P.libani* | Lebanon: Libani | Geiger et al. 2014 | ZFMK:FSJF:2154 |
| KJ554422 | *P.libani* | *P.libani* | Lebanon: Libani | Geiger et al. 2014 | ZFMK:FSJF:2154 |
| KJ554436 | *P.libani* | *P.libani* | Lebanon: Libani | Geiger et al. 2014 | ZFMK:FSJF:2154 |
| KJ554469 | *P.libani* | *P.libani* | Lebanon: Libani | Geiger et al. 2014 | ZFMK:FSJF:2154 |
| MK370409 | *P.turani* | *P.turani* | Turkey: Orontes | Geiger, Unpublished | FSJS-DNA-334 |
| HM560312 | *P.turani* | *Pseudophoxinus cf. kervillei* | Turkey: Incesu spring, Hassa | Perea et al. 2010 | MNCN AT5284 |
| KJ554335 | *P.turani* | *Pseudophoxinus sp.* | Turkey: Orontes | Geiger et al. 2014 | ZFMK:FSJF:1379 |
| KJ554397 | *P.turani* | *Pseudophoxinus sp.* | Turkey: Orontes | Geiger et al. 2014 | ZFMK:FSJF:1379 |
| KJ554484 | *P.turani* | *Pseudophoxinus sp.* | Turkey: Orontes | Geiger et al. 2014 | ZFMK:FSJF:334 |
| KJ554186 | *P.zeregi* | *P.zeregi* | Syria: Abu Noah | Geiger et al. 2014 | ZFMK:FSJF:1157 |
| KJ554229 | *P.zeregi* | *P.zeregi* | Syria: Abu Noah | Geiger et al. 2014 | ZFMK:FSJF:1233 |
| KJ554387 | *P.zeregi* | *P.zeregi* | Syria: Abu Noah | Geiger et al. 2014 | ZFMK:FSJF:1233 |
| KJ554518 | *P.zeregi* | *P.zeregi* | Syria: Orontes | Geiger et al. 2014 | ZFMK:FSJF:1320 |
| HM560316 | *P.zeregi* | *P.zeregi* | Turkey: Buyuk | Perea et al. 2010 | MNCN AT5297 |
| HM560317 | *P.zeregi* | *P.zeregi* | Turkey: Buyuk | Perea et al. 2010 | MNCN AT5298 |
| KJ554459 | *P.zeregi* | *P.zeregi* | Turkey: Orontes | Geiger et al. 2014 | ZFMK:FSJF:377 |
| MK370418 | *P.zeregi* | *P.zeregi* | Turkey: Queiq | Geiger, Unpublished | FSJF-DNA-1382 |
