# Supplementary material for: ﻿A new Pseudophoxinus species (Teleostei, Cypriniformes, Leuciscidae) from the upper Jordan River basin (Israel) with comments on the status of a few other congeneric species
Source: Zookeys. 2025 Aug 25;1249:303–15. doi: 10.3897/zookeys.1249.154110 (PMC12402820; doi:10.3897/zookeys.1249.154110)
Supplement: Supplementary material 3 — Selected meristic counts and body proportions of non-type specimens of P. galilaeus [file zookeys-1249-303_article-154110__-s003.docx]

|  | N | Range | Average | S.D. |
| --- | --- | --- | --- | --- |
| **Counts** |  |  |  |  |
| TL | 178 | 25.6-81.9 | 46.9 | 12.5 |
| SL | 178 | 21-69.3 | 38.4 | 10.9 |
| No. of scale series along the body | 148 | 40-44 | 43.0 | 1.0 |
| Predorsal scales | 148 | 19-23 | 21.0 | 1.0 |
| Dorsal fin | 148 | I 7-8 | 7.6 | 0.5 |
| Anal fin | 148 | I 7-8 | 7.5 | 0.5 |
| Pectoral fin | 156 | 13-15 | 14.0 | 0.5 |
| Pelvic fin | 156 | 6-7 | 6.6 | 0.2 |
| Caudal fin rays | 138 | 20-24 | 21.1 | 0.8 |
| GR | 15 | 6-7 | 6.6 | 0.0 |
|  |  |  |  |  |
| **Proportions (%)** |  |  |  |  |
| SL/TL | 178 | 83.2-87 | 85.1 | 1.8 |
| Head length /SL | 178 | 27.1-33.7 | 27.7 | 1.7 |
| Body depth /SL | 178 | 25.9-32.8 | 25.2 | 2.2 |
| Eye diameter of head length | 148 | 26-32 | 27.1 | 2.8 |
| Depth of caudal peduncle of SL | 148 | 9-13.9 | 12 | 1.2 |
| Length of caudal peduncle of SL | 148 | 17.7-23 | 20.3 | 1.4 |
| Interorbital space of head length | 148 | 32-43.4 | 38.8 | 2.4 |
| Longest dorsal ray of SL | 148 | 10.3-28.9 | 19.2 | 4.6 |
| Longest anal ray of SL | 148 | 12-17.2 | 15.4 | 1.7 |
| Pectoral fin length of SL | 148 | 12.5-20.4 | 15.2 | 1.7 |
| Pelvic fin length of SL | 148 | 44.8-55 | 13.9 | 1.9 |
| Distance snout – pelvic fin of SL | 148 | 44.3-54.2 | 48.3 | 2.4 |
| Distance snout – pectoral fin of SL | 148 | 24.8-332.6 | 29.1 | 2 |
| Distance snout – dorsal fin of SL | 148 | 52.5-61.1 | 55 | 1.8 |
| Distance snout – anal fin of SL | 148 | 67.6-71 | 66.8 | 1.5 |
| Length of dorsal-fin base of SL | 148 | 10.9-14 | 12.5 | 0.6 |
| Length of anal-fin base of SL | 148 | 10.2-13 | 11.3 | 0.9 |

Table S2. Selected meristic counts and body proportions of non-type specimens of *P. galilaeus*
